# Knowledge, Attitude, and Practice of House Officers to the Diagnosis and Management of Bacterial Pharyngitis: A Multicenter Cross-Sectional Study

**DOI:** 10.7759/cureus.31872

**Published:** 2022-11-24

**Authors:** Muaz Hassan

**Affiliations:** 1 Medicine, Alnau Teaching Hospital, Khartoum, SDN

**Keywords:** rheumatic heart disease, rheumatic fever, penicillin g, pharyngitis, pediatric

## Abstract

Background: Pharyngitis is one of the most common clinical presentations in pediatric clinics. Streptococcal pharyngitis reaches a nadir in the early school years, and it is vital to diagnose it early to avoid the serious complications that can arise from improper treatment, such as acute rheumatic fever and rheumatic heart disease.

Materials and Methods: This is a cross-sectional multicenter study of medical house officers conducted from January to February 2018.

Results: This study surveyed 135 house officers at Khartoum's primary health centers and hospitals. Most of the respondents were males (76/135; 56%). Most of the house officers reported seeing patients with throat complaints 1-2 times per week (58/135; 43%). The most common complication was acute rheumatic fever (65/135; 48%). Out of all the criteria of a Group A beta-hemolytic streptococcus (GAS) pharyngitis diagnosis, absence of cough and tender lymph nodes were the least recognized by house officers, i.e. 11.9% (16/135) and 45.2% (61/135), respectively. Only (4/135) 3% knew the full criteria for diagnosing bacterial pharyngitis. Throat culture was the most commonly used lab test to diagnose bacterial pharyngitis (115/135; 85.2%). The majority of participants did not recognize an IM injection of benzathine penicillin G as the first-line management of bacterial pharyngitis (101/135; 74.8%). The participants had moderate knowledge (93/135; 68.9%). Those with poor knowledge also happened to have poor practice (p = 0.009). Those who see more cases per week were also more likely to have good knowledge (p = 0.000). House officers with a moderate attitude constituted about 48.9% (66/135) of the whole sample. The most often prescribed medication was amoxicillin-clavulanic acid for one week (53/135; 39.3%). The practice score was associated with a lower knowledge (p = 0.009). Only 20.7% (28/135) of the participants in our study were adherent to the use of benzathine penicillin G as the first-line management of bacterial pharyngitis.

Conclusion: House officers had poor-to-moderate knowledge regarding the diagnosis and management of bacterial pharyngitis, especially in regard to what antibiotic to use. However, their fear of antibiotic resistance was a good behavior.

## Introduction

Pharyngitis is one of the most common clinical symptoms of pediatrics and is also known as a sore throat. It is associated with infection/inflammation of the throat and tonsils. It can be caused by both bacterial and viral pathogens. Group A beta-hemolytic streptococcus (GAS) is the leading cause of bacterial pharyngitis worldwide, including in Sudan, accounting for 25% of pediatric patients with sore throat [1-4]. Distinguishable bacterial causes of pharyngitis rather than GAS include *Mycoplasma pneumoniae* (1%), group C Streptococci (5% of all cases), *Chlamydia pneumoniae* (1%), and anaerobic species (1%). Among the viruses, rhinovirus, coronavirus, and adenovirus account for 30% of all cases, while the Epstein virus accounts for 1%, and influenza and parainfluenza viruses account for about 4% [5].

Streptococcal pharyngitis peaks in early life, but is rare before age 3. The disease is most common in spring and winter [4]. The transmission of this infection occurs via respiratory droplets and it has an incubation period of 25 days. Transmission of infection is highest in the acute phase and gradually declines over several weeks in untreated people, and ceases 24 hours after antibiotic therapy [6].

Complications of GAS pharyngitis can be divided into purulent and nonpurulent. Peritonsillar abscess, cervical lymphadenitis, posterior pharyngeal abscess, sinusitis, otitis media, and mastoiditis can occur due to the spread of GAS to adjacent tissues. These complications can be reduced by the use of antibiotics. This remains a reality even if the primary disease is overlooked or untreated. Acute rheumatic fever (ARF), acute streptococcal glomerulonephritis, reactive arthritis, Sydenham's chorea, and pediatric autoimmune neuropsychiatric disorders are nonpurulent immune-mediated consequences associated with *Streptococcus pyogenes* [4].

A study conducted in 2014 found that the prevalence of rheumatic heart disease (RHD) in children over the age of 14 is high in sub-Saharan Africa (5.7 in 1000), indigenous people in southern Central Asia (2.2 in 1000), New Zealand and Australia (3.5 in 1000) and is far less in developed countries (usually 0.5 per 1000) [7]. In many countries, the combination of increased awareness and integration of primary and secondary prevention through early diagnosis and management leads to the eradication of RHD [7,8]. The main problem with RHD prevention is how difficult it is to confirm the diagnosis of GAS by throat culture or rapid antigen testing. Over the last 40 years, many clinical prediction rules (CPRs) have been developed to differentiate streptococcal pharyngitis from other causes of pharyngitis to improve proper noncultured formulations of antibiotics in all patients. CPR is an evidence-based tool that allows clinicians to categorize patients according to their likelihood of suffering from a particular disability. They can also be used as a reasonable basis for treatment [9-11]. The predicted value for this CPR is 36%, which approaches 50%, given the false-negative rate of 10-15% for throat cultures [12].

The most common CPR for GAS pharyngitis is the Centor score [13,14]. The Centor score consists of four signs and symptoms (fever, tonsillar exudate, tender cervical lymph node, absence of cough) and is recommended in clinical guidelines from the American College of Physicians-American Society of Internal Medicine (ACP-ASIM) and the Centers for Disease Control and Prevention (CDC) in the US. The ACP-ASIM recommends 1) empirical treatment of adults with all four criteria, rapid antigen detection tests of patients with two or three criteria, and further treatment of those with positive test results and no treatment for all others; 2) empirical antibiotic treatment of adults with at least three of the four Centor criteria and no treatment for all others [14].

The clinical scoring system is designed to predict the potential for streptococcal infection in children and adults with a sore throat. These systems are based on an assessment of suggestive clinical findings such as fever, swelling or exudate of the tonsils, tenderness and enlargement of the anterior cervical lymph nodes, and lack of cough. The likelihood of a positive result from a throat culture or rapid antigen test ranges from less than 3% of patients with no evidence of clinical standards to approximately 30-50% of all patients [13]. Prevention of GAS also prevents post-infection complications such as rheumatic fever, glomerulonephritis chain, and scarlet fever [14,15]. Also, one of the main scoring systems is the Centor score (correction/McIsaac). It predicts the potential for streptococcal infection in children and guides testing and treatment options. It consists of several elements like fever above 38°C and tonsillar exudate, each representing a point. Antibiotic treatment is not indicated for people with a score of 1 or less, but testing is recommended for people with a score of 2 or higher [16]. Local rules for diagnosis and management could also be used, and one of the local guidelines is Sudan's guidelines for the diagnosis and management of acute pharyngotonsillitis [17].

Treatment of other causes of pharyngitis is not justified as treatment of GAS pharyngitis, which emphasizes the importance of sufficient knowledge of diagnostic guidelines [16].

Antibiotics are used as a treatment option to decrease the risk of complications such as rheumatic fever, bacteremia, acute glomerulonephritis, and peritonsillar abscess. Antibiotics are also effective in reducing the duration of symptoms and the dissemination of the disease. However, antibiotics have been overprescribed, which led to several efforts to try to decrease the unnecessary use of antibiotics for respiratory tract infections in children including patient education and delayed prescription of antibiotics [3]. It is important to mention that antibiotics are usually prescribed according to clinical presentations of the patients such as sore throat, fever, and earache; it is also prescribed for other factors not related to the signs and symptoms of the patient such as the lack of national or local diagnostic and treatment guidelines [18,19].

Antibiotics are improperly prescribed by doctors to treat patients with a sore throat, regardless of the causative agent. Improper use of antibiotics raises concerns about microbial resistance and emphasizes the need for clear diagnostic clinical guidelines [20].

This study aimed to investigate house officers' knowledge, attitudes, and practices regarding clinical management guidelines for streptococcal pharyngitis.

## Materials and methods

This descriptive cross-sectional study was performed from January to February 2018 to determine the knowledge, attitude, and practice toward streptococcal pharyngitis management among children in primary healthcare centers and hospitals. The study was conducted at the following primary healthcare centers and hospitals:

1. Soba Hospital, which was established in 1975, is the main training hospital for medical students, postgraduate students, nursing college students, medical laboratory science students, and others.

2. Ibrahim Malik Hospital, established in 1977 and located in Al Sahafa, offers different medical specialties and is recognized by the Sudan Board of Medical Specialists for training registrars and house officers.

3. Omar Ibn Al Khattab Primary Health Care Center, which was established in 1989, is located in Arquit.

4. Ans Omdurman Hospital, which was established in 1898, consists of many specialties.

The study was conducted among the house officers who were attending these primary healthcare centers and hospitals. The sample size was calculated using the following formula:

N = n/1 + (nd2) = 133 where N = sample size, n = population size, and D = desired margin of error, D = 0.05.

The data were collected through a self-administered questionnaire using multistage systematic random sampling. The questionnaire was developed after extensive literature searching and reviewing. It consists of three parts about the knowledge, attitude, and practice regarding streptococcal pharyngitis management. The scoring system of knowledge and practice is shown in the Results section.

Statistical analysis

The data used in this study were analyzed using statistical package for social sciences (SPSS) version 24.0 (IBM Corp, Armonk, NY) and presented using frequency and tables.

Ethical consideration

Verbal consent was obtained from each participating person.

## Results

This study surveyed 135 house officers at Khartoum's primary health centers and hospitals; 56% (76/135) of participants were males; 43.0% (58/135) saw a child with a sore throat complaint in an outpatient clinic every 1-2 weeks; 8.1% (11/135) did not see a child with a sore throat; and 48.1% (65/135) encountered ARF as a complication of bacterial pharyngitis in their clinical practices (Table 1). 

**Table 1:** Frequency of Children Presenting With Complications

Variable	Number	Percent
Males	76	56
Females	59	44
How often do you see a child with a sore throat complaint in your outpatient clinic?		
No patient	11	8.1
1-2/week	58	43
3-5/week	37	27.4
More than 5	26	19.3
What complication of bacterial pharyngitis have you encountered in your clinical practice?		
Local complication: abscess, quinsy		
Yes	48	35.6
No	87	64.4
Acute rheumatic fever		
Yes	65	48.1
No	70	51.9
I did not see a patient with complications		
Yes	48	35.6
No	87	64.4

Of all the participants, 3.0% (4/135) knew all clinical criteria for diagnosing bacterial pharyngitis. 2% (3/135) did not know the clinical criteria for the diagnosis. 80.7% (109/135) could differentiate viral from bacterial pharyngitis by fever and tonsillar exudate. 85.2% (115/135) knew that a throat culture is a lab test that can support bacterial pharyngitis diagnosis. 23.7% (32/135) knew that a rapid antigen test is a lab test that can support bacterial pharyngitis diagnosis. 25.0% (34/135) knew that a single IM injection of benzathine penicillin G is the best management of bacterial pharyngitis, according to the Sudan protocol for sore throat management. 41.5% (56/135) knew that taking macrolides for 10 days is the second-line management of bacterial pharyngitis, according to the Sudan protocol for sore throat management. 63.0% (85/135) knew that ARF is the most common systemic complication in bacterial tonsillitis in Sudan. 23.7% (32/135) had good knowledge and 68.9% (93/135) had moderate knowledge. The overall knowledge score mean was 6.4 and SD =1.5 (Table 2).

**Table 2:** Knowledge Regarding Streptococcus Pharyngitis Management in Children Among House Officers ESR, erythrocyte sedimentation rate; ASO, anti-streptolysin O.

Variable		Number		Percent
What are the clinical criteria for diagnosis of bacterial pharyngitis?				
Fever				
Yes		119		88.1
No		16		11.9
Tonsillar exudate				
Yes		109		80.7
No		26		19.3
Tender cervical lymph node				
Yes		61		45.2
No		74		54.8
Absence of cough				
Yes		16		11.9
No		119		88.1
Presence of cough				
Yes		92		68.1
No		43		31.9
I don't know				
Yes		3		2.2
No		132		97.8
Know all clinical criteria for diagnosing bacterial pharyngitis				
Fever, tonsillar exudate, tender cervical lymph node, absence of cough		4		3.0
How to differentiate viral from bacterial pharyngitis?				
High-grade fever and exudate in tonsils occur in bacterial type		109		80.7
High total white blood cell count		18		13.3
There is no way to differentiate		3		2.2
I do not know		5		3.7
What are the lab tests that can support bacterial pharyngitis diagnosis?				
ESR				
Yes		35		25.9
No		100		74.1
ASO titer				
Yes		92		68.1
No		43		31.9
Rapid antigen test				
Yes		32		23.7
No		103		76.3
Throat culture				
Yes		115		85.2
No		20		14.8
I do not know				
Yes		4		3.0
No		131		97.0
Rapid antigen test + throat culture		30		22.2
What is the management of bacterial pharyngitis according to the Sudan protocol for sore throat management?				
3 daily doses of azithromycin-Zomax		41		30.1
single IM injection of benzathine penicillin G		34		25.0
Amoxicillin and clavulanic acid combination for 7 days		40		29.4
I do not know		14		10.4
If sensitive to first-line treatment, what is the second line according to the Sudan protocol for sore throat management?				
Macrolides for 10 days		56		41.5
Benzylpenicillin for 7 days		37		27.4
Ciprofloxacin for 7 days		9		6.7
No other medication is effective in eradication of bacterial (GAS) tonsillitis		1		0.7
I do not know		28		20.7
What is the most common systemic complication of bacterial tonsillitis in Sudan?				
Glomerulonephritis		18		13.3
Acute rheumatic fever		85		63.0
Only local complications like abscess with no systemic effect		5		3.7
I do not know		10		7.4
Wrong answers		17		12.6
Knowledge categories				
Poor	10		7.4	
Moderate	93		68.9	
Good	32		23.7	
Knowledge score				
Mean	6.4148			
SD	1.54719			

Using a chi-square test, good knowledge was significantly associated with good practice (p = 0.009), ARF as a complication of bacterial pharyngitis (p = 0.000), and frequency of seeing a child with sore throat in outpatient clinic (p = 0.000) (Table 3).

**Table 3:** Cross-Tabulation of Factors Affecting Respondents’ Knowledge Regarding Streptococcus Pharyngitis Management in Children

Variables	Knowledge categories			Chi-square	p-Value
	Poor	Moderate	Good		
Gender					
Male	5	54	17	0.808	0.128
Female	5	39	15		
Attitude categories					
Poor	2	15	6	0.277	0.063
Moderate	3	46	17		
Good	5	32	9		
Practice categories					
Poor	7	62	13	0.018	0.009
Moderate	3	26	17		
Good	0	5	2		
What complications of bacterial pharyngitis have you encountered in your clinical practice?					
Local complication: abscess, quinsy	3	32	13	0.796	0.102
Acute rheumatic fever	2	37	26	0.000	0.000
Frequency of seeing child with sore throat in outpatient clinic					
No patient	2	9	0	0.041	0.000
1-2/week	6	42	10		
3-5/week	0	24	13		
More than 5	1	16	9		

In our sample, 72.6% (98/135) thought bacterial pharyngitis should be diagnosed by a combination of clinical and laboratory criteria. 60.7% (82/135) preferred the prescription of antibiotics to cases confirmed to be bacterial pharyngitis. 56.3% (76/135) preferred giving antibiotics after culture and sensitivity tests and 10.4% (14/135) preferred giving a benzathine penicillin G injection. 48.9% (66/135) had a moderate attitude. The overall attitude score mean was 2.1 (SD = 0.76). 

There is a significant association between the attitudes and encountering local complications as a complication of bacterial pharyngitis (p = 0.042) (Table 4).

**Table 4:** Cross-Tabulation of Factors Affecting Respondents’ Attitude Regarding Streptococcus Pharyngitis Management in Children

Variables	Attitude categories			Chi-square	p-Value
	Poor	Moderate	Good		
Gender					
Male	15	37	24	0.605	0.062
Female	8	29	22		
Knowledge categories					
Poor	2	3	5	0.740	0.063
Moderate	15	46	32		
Good	6	17	9		
Practice categories					
Poor	15	36	31	0.572	0.066
Moderate	7	25	14		
Good	1	5	1		
What complications of bacterial pharyngitis have you encountered in your clinical practice?					
Local complication: abscess, quinsy	9	27	12	0.258	0.042
Acute rheumatic fever	6	40	19	0.008	0.088
Frequency of seeing child with sore throat in outpatient clinic					
No patient	2	5	4	0.511	0.051
1-2/week	9	27	22		
3-5/week	5	21	11		
More than 5	5	13	8		

Of the study participants, 43.7% (59/135) diagnosed bacterial pharyngitis by clinical examination of the pharynx. 62.2% (84/135) could differentiate between viral and bacterial pharyngitis. 20.7% (28/135) gave a single IM injection of benzathine penicillin G. 60.7% (82/135) had poor practice. The overall practice score mean was 1.3 (SD = 0.81) (Table 5).

**Table 5:** Practice Regarding Streptococcus Pharyngitis Management in Children Among House Officers ESR, erythrocyte sedimentation rate; ASO, anti-streptolysin O.

Variable	Number	Percent
How do you diagnose bacterial pharyngitis?		
I depend on my examination of the pharynx	59	43.7
I depend on high total WBCs in complete blood count (CBC)	22	16.3
I order ASO, ESR, and manage accordingly	24	17.8
It is difficult to examine the throat of a child, so I start treatment for every sore throat with fever	10	7.4
Wrong answers	20	14.8
Can you clearly differentiate between viral and bacterial pharyngitis?		
Yes	84	62.2
No	51	37.8
What medication do you often describe to patients with bacterial pharyngitis?		
I give single IM injection of benzathine penicillin G	28	20.7
I give 3 days' course of azithromycin	47	34.8
I give 1-week course of amoxicillin-clavulanic acid combination (Amoclan)	53	39.3
I advise mother to give warm drinks and prescribe antipyretic only	3	2.2
Practice categories		
Poor	82	60.7
Moderate	46	34.1
Good	7	5.2
Practice score		
Mean	1.2667	
SD	0.8122	

There were significant associations between good practice and good knowledge (p = 0.009) and ARF as a complication of bacterial pharyngitis (p = 0.007) (Table 6).

**Table 6:** Cross-Tabulation of Factors Affecting Respondents’ Practice Regarding Streptococcus Pharyngitis Management in Children

Variables	Knowledge categories			Chi-square	p-Value
	Poor	Moderate	Good		
Gender					
Male	42	30	4	0.372	0.055
Female	40	16	3		
Knowledge categories					
Poor	7	3	0	0.088	0.009
Moderate	62	26	5		
Good	13	17	2		
Attitude categories					
Poor	15	7	1	0.572	0.066
Moderate	36	25	5		
Good	31	14	1		
What complications of bacterial pharyngitis have you encountered in your clinical practice?					
Local complication: abscess, quinsy	26	21	1	0.169	0.105
Acute rheumatic fever	33	27	5	0.051	0.007
Frequency of seeing child with sore throat in outpatient clinic					
No patient	8	3	0	0.330	0.059
1-2/week	34	18	6		
3-5/week	20	16	1		
More than 5	17	9	0		

## Discussion

This cross-sectional study aimed to explore house officers' knowledge, attitude, and practices regarding the diagnosis and management of GAS pharyngitis in children in Khartoum state. The majority of respondents were males (76/135; 56%). Most of the house officers saw patients with throat complaints 1-2 times per week (58/135; 43%). The most common complication that was seen was ARF (65/135; 48.1%). Out of all the criteria of GAS pharyngitis diagnosis, the absence of cough and the presence of tender cervical lymph nodes were the least recognized by house officers, i.e. 11.9% (16/135) and 45.2% (61/135), respectively. Only 3% (4/135) knew the full criteria for diagnosing bacterial pharyngitis. Throat culture was the most commonly used lab test to diagnose bacterial pharyngitis (115/135; 85.2%). The majority of patients did not recognize IM injection of benzathine penicillin G as the first-line management of bacterial pharyngitis (101/135; 74.8%). The majority also did not recognize macrolides as the second line of treatment (79/135; 58.5%). Overall, the participants had moderate knowledge (93/135; 68.9%). Those with poor knowledge are also suggested to have poor practice (p = 0.009). Those who see more cases per week were also more likely to have more knowledge (p = 0.000). These findings could be explained by poor knowledge of Sudan's guidelines for the diagnosis and management of acute pharyngotonsillitis [17].

Most house officers thought that both clinical and laboratory testing should be used to diagnose bacterial pharyngitis (98/135; 72.6%). Most house officers (82/135; 60.7%) were reserved about antibiotic use in only cases with a confirmed diagnosis. This reflects a good awareness of the risks of antibiotic resistance and the complications that may arise from it in our house officers in this study. About 56% (76/135) of the house officers thought that the use of an antibiotic is dependent on the culture sensitivity test to choose the best one. This also reflects a good perception of drug resistance. House officers with a moderate attitude constituted about 49% (66/135) of the whole sample.

Most of the house officers relied on examination of the pharynx to determine the diagnosis of bacterial pharyngitis (59/135; 43.7%). Most of them also said they were able to differentiate between viral and bacterial pharyngitis. The most often prescribed medication was amoxicillin-clavulanic acid for one week (53/135; 39.3%). Practice score was associated with lower knowledge (p = 0.009). 

The Centor score could be given different weights depending on whether the physician wants to rule in or out the diagnosis of GAS pharyngitis. This is aided by the finding that the signs of fever and "any exudate" have a higher specificity than sensitivity tests and are more valid for ruling in a diagnosis. On the other hand, the signs of tender anterior cervical lymphadenopathy and absence of cough have a higher sensitivity than specificity and are more valid for ruling out GABHS pharyngitis when absent [3]. 

Only 3% of the house officers in our study knew the full criteria to diagnose bacterial pharyngitis. Only 20.7% of the participants in our study were adherent to the use of benzathine penicillin G as the first-line management of bacterial pharyngitis. This is similar to another study that had an adherence rate of 27.7% [20]. This is also less than that in another study, which had an adherence rate of 34% [21]. Some others had even lower adherence to the indication of treatment (16%) in community physicians.

The diagnostic accuracy of C-reactive protein (CRP) is dependent on the presence of not a single sign or symptom but a number. The presence of a single sign or symptom on its own is not enough to rule in or out a diagnosis [22]. Other studies, however, have found that these signs and symptoms have different predictive values. One study, for example, found that among the four Centor criteria, only the absence of cough and cervical lymphadenitis were significantly more frequent in the GAS pharyngitis patients compared to those with negative cultures [23,24]. Another study found that tonsillar exudate had no predictive ability [25].

A barrier when introducing CPRs such as the Centor score into practice is that clinicians often fail to apply them [23,26]. One community-based study that used repeated clinical prompts for the modified Centor score to try to influence physicians’ behavior when prescribing antibiotics for sore throats found no significant change in physician behavior. However, the authors had problems retaining community-based physicians for the duration of the study and believe their results may have been biased by these losses [26].

A study was conducted in Saudi Arabia to examine the adherence of physicians to the guidelines for diagnosing GAS pharyngitis. About 2/3 (66.1%) of the participants were between the ages of 25 and 35 and half of them were male. Regarding the knowledge score of the participants, 9.1% of them gained a score of 0, while 46.5% scored 1 and 44.4% scored 2 (a perfect score). When it comes to adherence to the guidelines, only 27.7% were adherent, while the majority were nonadherent. Of those who adhered to the guidelines, 70.9% explained that they were adherent because it was the guideline recommendations, while 17.7% were conforming to the hospital policy. The remaining 11.8% followed the guidelines because of the insufficiency of the signs and symptoms. As for the nonadherent group, the most common rationale behind not following the guidelines was the claim that "symptoms and signs are satisfactory to make the diagnosis of acute bacterial pharyngitis." This was followed by difficulties in ensuring proper follow-up of patients and parental pressure to prescribe antibiotics, respectively. The unavailability of diagnostic tests is the least of all factors behind suboptimal adherence [20].

An Australian study explored the adherence of general practitioners (GPs) to guidelines for the treatment of GAS pharyngitis in pediatric patients. Most (89.9%) of the GPs would recommend antibiotic treatment if the patient’s clinical presentation was suggestive of a sore throat. Almost all the GPs recommended symptomatic treatment in isolation or conjunction with antibiotics (94%). Paracetamol was the most frequently recommended symptomatic treatment (88%), followed by nonsteroidal anti-inflammatory drugs (43%) [26].

Study limitations

The sample size was small because the samples were collected for a shorter time period only and the sample size could have been greater if it had been collected for an extended period of time. The other limitations of the study are the lack of finances and human resources.

Strength of the study

It covers three major hospitals in the capital of Sudan with high facilities and high capacity of patients and it gives a clue about the practice regarding diagnosis and management of streptococcal pharyngitis which helps in decreasing the incidence of complications.

## Conclusions

House officers had poor-to-moderate knowledge regarding the diagnosis and management of bacterial pharyngitis, especially regarding what antibiotic to use. However, their fear of antibiotic resistance was good behavior.
